# Electrodeposited PEDOT:PSS-Al_2_O_3_ Improves the Steady-State Efficiency of Inverted Perovskite Solar Cells

**DOI:** 10.3390/polym13234162

**Published:** 2021-11-28

**Authors:** Eider A. Erazo, Martín Gómez, Leonardo Rios, Edgar J. Patiño, María T. Cortés, Pablo Ortiz

**Affiliations:** 1 Departamento de Química, Universidad de los Andes, Bogotá D.C. 111711, Colombia; ea.erazo@uniandes.edu.co; 2 Departamento de Ingeniería Química, Universidad de los Andes, Bogotá D.C. 111711, Colombia; m.gomezd@uniandes.edu.co (M.G.); portiz@uniandes.edu.co (P.O.); 3 Superconductivity and Nanodevices Laboratory, Departamento de Física, Universidad de los Andes, Bogotá D.C. 111711, Colombia; l.rios954@uniandes.edu.co (L.R.); epatino@uniandes.edu.co (E.J.P.)

**Keywords:** PEDOT:PSS, perovskite solar cell, electrodeposited HTM, Al_2_O_3_

## Abstract

The atomic layer deposition (ALD) of Al_2_O_3_ between perovskite and the hole transporting material (HTM) PEDOT:PSS has previously been shown to improve the efficiency of perovskite solar cells. However, the costs associated with this technique make it unaffordable. In this work, the deposition of an organic–inorganic PEDOT:PSS-Cl-Al_2_O_3_ bilayer is performed by a simple electrochemical technique with a final annealing step, and the performance of this material as HTM in inverted perovskite solar cells is studied. It was found that this material (PEDOT:PSS-Al_2_O_3_) improves the solar cell performance by the same mechanisms as Al_2_O_3_ obtained by ALD: formation of an additional energy barrier, perovskite passivation, and increase in the open-circuit voltage (V_oc_) due to suppressed recombination. As a result, the incorporation of the electrochemical Al_2_O_3_ increased the cell efficiency from 12.1% to 14.3%. Remarkably, this material led to higher steady-state power conversion efficiency, improving a recurring problem in solar cells.

## 1. Introduction

The solution processing of organic–inorganic perovskite solar cells is a promising route for the fabrication of cost-effective photovoltaic devices [1,2]. These solar cells can be grouped into two main architectures: direct and inverted. They differ in the direction of electron flow within the device, flowing towards the conductive glass for the direct devices and towards the metal electrode for the inverted architecture. This requires the use of different charge extraction materials. In direct architecture devices, the light is incident on the conductive oxide and travels across a transparent electron-transporting material (ETM) before reaching the perovskite. For this architecture, the most commonly used materials are TiO_2_ as ETM, and Spiro-OMeTAD as HTM. However, high costs, high-temperature processes (450 °C), and hysteresis in cells made with these materials have led to the development of the inverted architecture. Unlike direct solar cells, inverted architecture features devices with the HTM being deposited over the conductive oxide, hence this material must be transparent to allow incident light to reach the active layer [3,4]. Research on HTMs for perovskite solar cells is currently very relevant, as important requirements have not yet been fulfilled. High hole mobility is needed to ensure fast charge transport and high chemical and electrical stability, and conducting this at a low cost is also necessary [5,6,7].

PEDOT:PSS is a conductive polymer widely used as HTM in inverted perovskite solar cells due to its high transparency, flexibility, and low commercial cost. One of the most significant benefits of this polymer is the variety of techniques available for its deposition, as it can be spin-coated, spray-coated, doctor-blade coated, and even electrodeposition coated [8]. Nevertheless, achieving high power conversion efficiency (PCE) using PEDOT:PSS is challenging, and one of the main causes is its poor energy alignment with the perovskite. On the one hand, the highest occupied molecular orbital (HOMO) for PEDOT:PSS is only about 5.1 eV, compared to the valence band of the perovskite layer (5.4 eV) [9], which leads to important losses in the open-circuit voltage (V_oc_) of the devices, causing a V_oc_ below 1 V [10,11]. On the other hand, PEDOT:PSS has a low lowest unoccupied molecular orbital (LUMO) compared to polymers such as PTAA, resulting in a poor electron-blocking ability [12]. Moreover, PEDOT:PSS in its conducting state exhibits polaron and bipolaron mid-gap states that further decrease its effectiveness in preventing charge recombination [13,14].

The addition of a thin insulating layer of Al_2_O_3_ between the perovskite and the HTM has proved to be an effective strategy to reduce charge recombination, as it allows the passage of holes by tunneling and generates a higher energy barrier for electrons [15,16,17]. Koushik et al. made direct architecture devices by depositing Al_2_O_3_ by atomic layer deposition (ALD) on the perovskite layer to protect it before depositing the PEDOT:PSS by spin-coating. The deposited layer did not only protect the perovskite, but also passivated it, resulting in suppressed charge recombination. Hence, with the Al_2_O_3_ interlayer, the PCE of the devices increased from 9.6 to 11.2% and the steady-state PCE from 4 to 6% [18]. In another work, the same authors reported that an ALD Al_2_O_3_ interlayer between the perovskite film and the HTM Spiro-OMeTAD improved both the dynamic and steady-state PCE of the cells, increasing from 15% to 18% [17]. The addition of the Al_2_O_3_ interlayer requires nanometer-accurate thickness adjustment, which is generally achieved by ALD [15,17,18]. Nevertheless, this technique has significant disadvantages, such as being very expensive, requiring high vacuum conditions, having a very high energy and material waste rate, and emitting nanoparticles [19]. An effective alternative to this is electrodeposition, a process that is carried out in a simple electrochemical setup, with low-cost, non-vacuum, and low waste.

In this work, we present similar improvements to those achieved by ALD in inverted PEDOT:PSS devices, but using an electrodeposited PEDOT:PSS/Al_2_O_3_ HTM bilayer. In our previous work, the electropolymerized PEDOT:PSS-Cl was presented as an alternative to the solution-processed PEDOT:PSS HTM in perovskite solar cells [20]. From there, other papers have electrodeposited PEDOT using dopants different to the PSS, and to date a 13.56% PCE has been demonstrated by optimizing the monomer concentration and the electric charge used in the electropolymerization [21,22]. In this work, we try to improve the performance of the electropolymerized PEDOT by depositing an Al_2_O_3_ overlayer through an electrochemical process as well. To the best of our knowledge, this is the first time that a layer of electrodeposited Al_2_O_3_ is used in perovskite solar cells.

In order to obtain the PEDOT:PSS-Cl/Al_2_O_3_ bilayer, which we will call PEDOT-Al, PEDOT:PSS-Cl was polymerized on an ITO substrate from an aqueous solution of EDOT, NaPSS, and NaCl. Subsequently, an Al_2_O_3_ film was deposited from an aqueous AlCl_3_ solution by applying a constant potential. Finally, the resulting film was thermally annealed and subsequently used as HTM in inverted perovskite solar cells. The Al_2_O_3_ layer generated improvements in the efficiency and steady-state efficiency of the cells due to the passivation of perovskite. In addition, the bilayer was found to generate an effective energy barrier and confer protection to the polymer from halides, thus maintaining its semiconducting state.

## 2. Materials and Methods

### 2.1. Electrodeposition of PEDOT:PSS-Cl

The electrodeposition of the HTM was performed as in our previous report [20]; the patterned ITO glass substrates were scrubbed with a soft brush and a cloth using an aqueous solution of neutral detergent. The substrates were then sequentially subjected to ultrasonic cleaning, once in the detergent solution, and twice in deionized water for 15 min each time. The substrates were dried with compressed air and then in an oven at 95 °C. After drying, the substrates were plasma cleaned for 15 min. The electrodeposition of PEDOT:PSS-Cl was performed in a three-electrode cell; the working electrode was an ITO substrate; a platinum wire was used as a counter-electrode, and Ag/AgCl (3 M NaCl) as the reference electrode. The synthesis solution contained 4.5 g/L NaPSS (Aldrich, Average Mw 70,000, St. Louis, MO, USA), 0.02% *v/v* EDOT (Aldrich, 97%, St. Louis, MO, USA), and 0.1 M NaCl (Panreac ≥ 99.5%, Darmstadt, Germany). This solution was bubbled with N_2_ for 5 min and electropolymerization was conducted by cyclic voltammetry between −0.1 and 1.2 V at 100 mVS^−1^ until a charge of 4 mC/cm^2^ was reached.

### 2.2. Electrodeposition of Al_2_O_3_ on PEDOT:PSS-Cl

Obtaining Al_2_O_3_ by electrodeposition requires thermal annealing to achieve the transition from Al(OH)_3_ [23,24]. A lower thickness facilitates the transition and on the nanometer scale, and it starts as low as 200 °C [25,26]. Thus, to obtain the PEDOT-Al bilayer, PEDOT:PSS-Cl substrates were used as working electrodes to which a potential of −1 V (vs Ag/AgCl) was applied in a 0.02 M AlCl_3_ aqueous solution (pH 4.2). As a result, an Al(OH)_3_ deposit was formed and the substrates were thermally annealed at 200 °C, 225 °C, and 240 °C for 1 h. To obtain nanometer scale deposits, the electrodeposition charge was set at 0.50 mC cm^−2^ and 0.75 mC cm^−2^.

### 2.3. Solar Cell Fabrication

To test the effect of Al_2_O_3_ on the performance of PEDOT:PSS-Cl as HTM, inverted perovskite solar cells were fabricated in a glovebox with a constant N_2_ flow. First, the ITO/PEDOT:PSS-Cl substrates were annealed at 110 °C for 10 min, then 30 µL of perovskite precursor solution were deposited. This solution was prepared by dissolving 1.2 mmol of PbI_2_ (99,9985%, Alfa Aesar, Haverhill, MA, USA) and 1.2 mmol of methylammonium iodide (Greatcellsolar, Queanbeyan, Australia) in 1 mL of a mixed solvent containing DFM and DMSO in a volumetric ratio of 10:1. Spin coating was started with a two-stage program, 10 s at 1000 rpm followed by 15 s at 5000 rpm. After 6 s of starting the second stage, 350 µL of chlorobenzene were added, and then the PCBM layer, which acts as ETM, was deposited by dynamic spin coating (25 µL of a 20 mg/mL solution in chlorobenzene at 5000 rpm for 30 s). Subsequently, the substrate was heated at 100 °C for 30 min, which is known as merged annealing [27]; the obtained perovskite layer was about 400 nm thick. Finally, the BCP layer was spin-coated from 40 µL of a 0.5 mg/mL solution in methanol:toluene 100:1 at 4000 rpm for 40 s. The PCBM/BCP layer was about 25 nm, the BCP helps to form an ohmic contact between PCBM and Ag, preventing direct contact between both materials [28]. The device was removed from the glovebox and taken to a thermal evaporator in which 100 nm of Ag were deposited by monitoring its thickness using a quartz sensor; the first 5 nm were deposited at a rate of 0.2 Å s^−1^, and, thereafter, the rate was kept at 1 Å s^−1^.

### 2.4. Characterization Methods

The solar cells were characterized with JV curves using AM 1.5G light (100 mWcm^−2^) with a solar simulator (Abet Technologies model 10500, Milford, CT, USA) in a potential range of −0.1 to 1.1 V at 50 mVs^−1^ (Autolab µ3AUT71099, Utrecht, The Netherlands). The light intensity was calibrated with a Hamamatsu S1133 photodiode, and the illuminated area of the devices was defined with a 0.065 cm^2^ black shadow mask. The steady-state efficiency was determined with an unencapsulated cell; first its efficiency (PCE) was calculated from the JV curve, then a constant voltage was found and applied to keep the efficiency at a maximum. The steady-state PCE was monitored over time under a N_2_ atmosphere. For stability during impedance spectroscopy (IS) measurements under a N_2_ atmosphere, the devices were encapsulated with hot glue [29]. An illumination of 1 sun with an AC signal of 0.075 V was used, and measurements were made up to 0.6 V DC [30]. UV-Vis spectra were taken on an Analytik Jena SPECORD 50 PLUS spectrophotometer (Jena, Germany) and PL measurements on a Cary Varian Eclipse Fluorescence Spectrophotometer with an excitation wavelength of 405 nm. Impedance measurements and electrochemical characterizations were performed on an Autolab AUT84194 potentiostat equipped with the FRA module. HOMO level estimation by cyclic voltammetry was performed in anhydrous acetonitrile (Sigma Aldrich, HPLC grade, Darmstadt, Germany) with 0.1 M TBAPF6 (Aldrich, 98%, St. Louis, MO, USA) at 100 mV s^−1^, using 1 mM ferrocene (Alfa Aesar, 99%, Haverhill, MA, USA) as a vacuum electrochemical reference. The potential was measured against an Ag/AgCl reference electrode using a double-junction chamber filled with 3 M of NaCl. Mott–Schottky data were taken by applying a 10 mV AC perturbation at 1000 Hz, and each applied DC potential point was held 10 s before taking the measurement. Electrochemical impedance spectroscopy (EIS) was performed at 205 mV DC (vs. Ag/AgCl) over a frequency range of 100 kHz to 0.1 Hz with a 10 mV AC perturbation. Raman spectra were obtained with an Xplora Horiba Scientific confocal Raman microscope (Kyoto, Japan) employing a 532 nm laser, and a height scan was performed to obtain a clear spectrum of the films. AFM images were taken with an Asylum Research microscope model MFP-3D-BIO in tapping mode. SEM images were taken with a Tescan Lyra 3 microscope (Brno, Czech Republic). Finally, contact angles were measured using an Attension theta model contact angle meter.

## 3. Results

### 3.1. Influence of PEDOT-Al Bilayer on the Performance of Inverted Perovskite Cells

**Figure 1:**
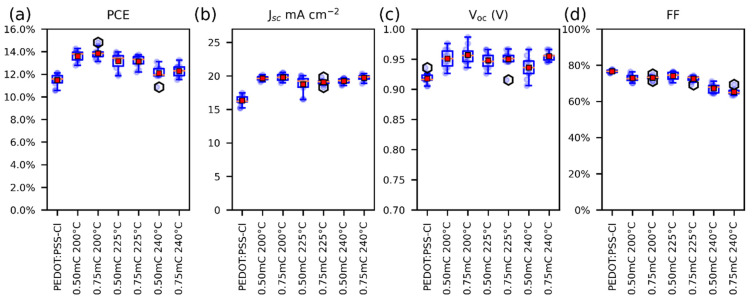
Box plots of the photovoltaic parameters for solar cells, (**a**) PCE, (**b**) J_SC_, (**c**) V_oc_, and (**d**) FF. For solar cells containing PEDOT-Al, only the variations are displayed on the labels. The red squares represent the mean and the orange lines the median (10 devices each variation).

The photovoltaic parameters of perovskite solar cells employing different PEDOT-Al layers as HTM were compared with the PEDOT:PSS-Cl reference device. To find suitable fabrication conditions for the Al_2_O_3_ layer, the annealing temperatures (200 °C, 225 °C, and 240 °C) and the electrodeposition charges per square centimeter (0.50 mC and 0.75 mC) were varied. The resulting photovoltaic parameters for each variation are illustrated in Figure 1 and summarized in Table S1. Improvements in power conversion efficiency (PCE), short current density (J_sc_), and open-circuit voltage (V_oc_) were observed for all cells in which the PEDOT-Al bilayer was used. It was found that the electrodeposition charge has no significant effect on the studied parameters, but the efficiency and fill factor (FF) of the cells decreased with increasing annealing temperature. Based on these results, it was determined that the best electrodeposition conditions for Al_2_O_3_ are a 200 °C annealing temperature and 0.50 mC charge. The latter was chosen for simplicity, as it optimizes the deposition time (around 6 s).

**Figure 2:**
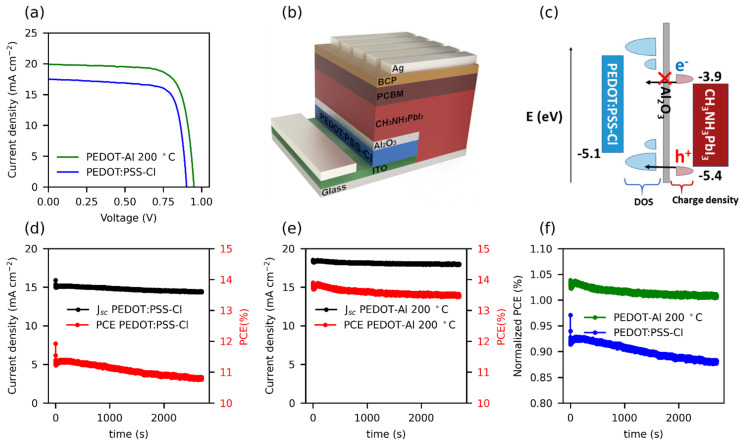
(**a**) JV curves of the best performing cells with PEDOT:PSS-Cl and PEDOT-Al 0.50 mC 200 °C. (**b**) Structure of the inverted perovskite solar cells containing the PEDOT-Al bilayer, and (**c**) schematic diagram of energy levels, including the DOS of PEDOT:PSS-Cl with its mid-gap states, and the charge density of electrons and holes for perovskite. (**d**,**e**) Stabilized current density and steady-state PCE under 1 sun illumination for (**d**) the reference cell with an initial JV efficiency (JV PCE) of 12.3%, and (**e**) the cell with PEDOT-Al 0.50 mC 200 °C with a 13.4% JV PCE. (**f**) Normalized steady-state PCE with respect to the JV PCE of each cell.

The reference solar cells reached a maximum efficiency of 12.1% (J_sc_: 17.5 mA cm^−2^, V_oc_: 0.905 V, and FF: 77%). In contrast, solar cells employing PEDOT-Al (0.50 mC, 200 °C) exhibited a maximum efficiency of 14.3% (J_sc_: 19.9 mA cm^−2^, V_oc_: 0.956 V, and FF: 75%) (Figure 2a). These results are lower than the 20.22% PCE reported for spin-coated CsI-PEDOT:PSS devices [31], but represent an improvement in the performance of electrodeposited PEDOT-based devices. As mentioned in the Introduction, the best PCE reported to date using an electrodeposited PEDOT-based MTH exhibits 13.56% (J_sc_: 22.19 mA cm^−2^, V_oc_: 0.94 V, and FF: 65%) [22]. In comparison, the results reported in this study correspond to a significantly higher FF, which compensates for the lower J_sc_, resulting in a higher PCE. Previously, the increased efficiency of solar cells using PEDOT/Al_2_O_3_ has been attributed to the improved charge selectivity [18]. Figure 2b shows the architecture used in this study, in which PEDOT has an energy configuration with a density of states (DOS) appropriate for hole extraction and unfavorable for electron transport. Therefore, holes have a higher probability of tunneling through the thin insulating layer than electrons (Figure 2c) [16,17].

A recurrent issue in perovskite solar cells is that the PCE measured from the JV curves (JV PCE) is not the actual steady-state power conversion efficiency maintained by the device [32]. Discrepancies between the PCE from the JV and the steady-state PCE are related to hysteresis in the JVs as well as to slow transient phenomena in the perovskite devices. The latter is connected to the ionic migration in the perovskite film, which causes changes in the internal electric field, charge recombination rates, and the interfacial trap states [33,34].

To measure the steady-state PCE, a constant potential of 0.75 V was applied to the cells for 45 min (see Figure 2d–f). When analyzing the first 200 s of the test, the PEDOT-Al 0.50 mC 200 °C cells demonstrated a slightly higher stabilized efficiency compared to their JV PCE, whereas PEDOT:PSS-Cl devices with an initial JV PCE of 12.3% were only able to maintain up to 92% of their JV PCE. Moreover, during the entire test, the steady-state PCE of the reference cell decreased by 4.2%, while the PEDOT-Al cell only had a loss of 2.2%. The trend of the curves in Figure 2f illustrates the significance of this result and the effect that the Al_2_O_3_ layer could have in the long term performance of the cells under continuous operation. The cells with PEDOT-Al also showed better shelf stability; after 25 days, the PEDOT:PSS-Cl devices maintained 75% of their initial FF, while the PEDOT-Al devices maintained 90% (Figure S1). The above meant that, for the PEDOT:PSS-Cl reference device, an average of 70% of its initial efficiency was maintained, compared to 77% for the PEDOT-Al devices. This also represents an advantage of PEDOT electrodeposition, since spin-coated PEDOT:PSS devices tend to degrade completely in about 20 days of storage [20,22].

A major drawback to obtain stable perovskite solar cells is the reactivity of the PEDOT:PSS/perovskite interface. First, it has been shown that methylammonium ions (MA^+^) can interact with PSS^−^ causing the PEDOT:PSS work function to decrease [35]. These ions tend to accumulate at this interface under dark conditions due to the built-in potential (V_bi_) of the solar cell [36]. Second, it has recently been observed that halide ions (I^−^, Cl^−^, and Br^−^) in the perovskite precursor solution dope the PEDOT:PSS [37], introducing mid-gap states leading to recombination [14,20]. Under illumination, near the maximum power point (V_mpp_) the net potential is reversed and I^−^ ions accumulate at the PEDOT:PSS/perovskite interface [36]. To test whether the Al_2_O_3_ layer could mitigate these deleterious effects on the PEDOT layer, a Mott–Schottky analysis of the HTMs in the presence of halide ions (Cl^−^) was performed. The tests were conducted by polarizing the polymer to provoke doping/dedoping processes.

The doping/dedoping effects induced by halide ions in PEDOT:PSS-Cl films are demonstrated by the Mott–Schottky plots (Figure S2a,b). The reverse scans (taken from 0.7 to −0.5 V) show a negative slope around −0.25 V, which is consistent with the p-doping of the polymer [38]. However, the negative slope was not observed after changing the scan direction (forward). To rule out that this trend was a product of polymer degradation, a third reverse scan was performed in which the slope reappeared, suggesting reversible behavior; the results were reproducible in several replicates. Consequently, this behavior can be attributed to the doping/dedoping process of the polymer. As for the PEDOT-Al bilayers, Mott–Schottky plots in Figure S2c–f for both scan directions show the expected negative slope for p-type polymers. This suggests that the electrodeposited inorganic layer, regardless of its deposition parameters, protects the polymer from being doped/dedoped. This agrees with the results obtained for steady-state PCE: a more constant steady-state efficiency for PEDOT-Al devices, which are protected from doping/dedoping effects, and an efficiency that decreases rapidly with time for reference cells, where the polymer is exposed to I- ions that induce changes in its electrical properties (Figure 2f) [36].

**Figure 3:**
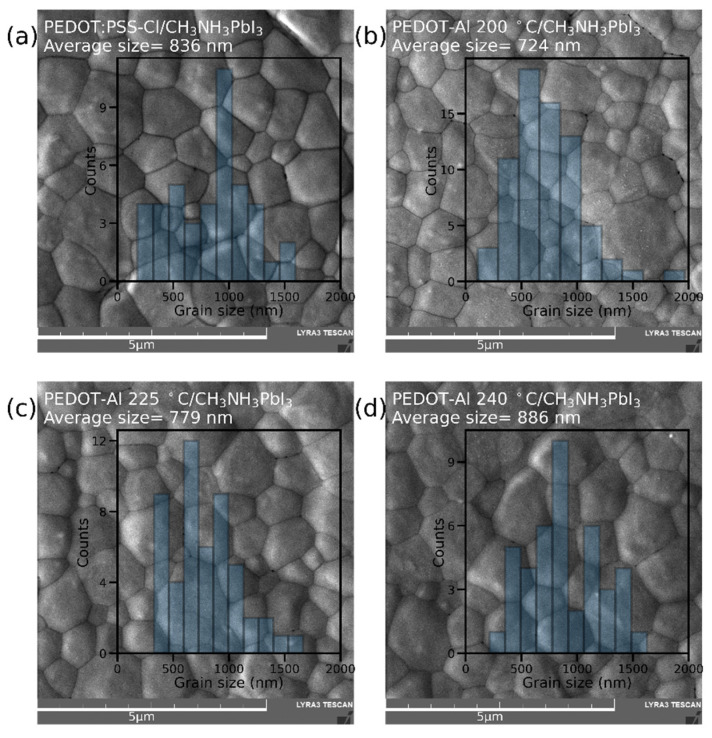
SEM top-view images and grain size distribution of perovskite films deposited on (**a**) PEDOT:PSS-Cl and PEDOT-Al 0.50 mC annealed at (**b**) 200 °C, (**c**) 225 °C, and (**d**) 240 °C. The scale bar is 5 µm.

The effect of PEDOT-Al 0.50 mC annealing temperature on perovskite film morphology was studied through top-view SEM images (Figure 3). Grains up to ~1.5 μm were found in all variations, possibly due to the merged annealing method that produces large-grain-size perovskite films [27]. Furthermore, when analyzing the average grain size, no trend was detected to explain the higher efficiencies with PEDOT-Al. The average grain size for annealing at 200 °C is even smaller than that of the reference device. This shows that the reason for the efficiency increase is not related to the perovskite grain size [39]. It can also be observed that the grain size distribution is above the perovskite layer thickness (400 nm) for all studied devices (Figure S3). Variations within these ranges do not significantly increase the grain boundaries in the vertical direction and do not induce larger recombination sites.

Figures S3 and S4 correspond to cross-sectional SEM images, and the thickness of the HTM layers were: PEDOT:PSS-Cl (7–10 nm), PEDOT-Al 0.50 mC 200 °C (10–13 nm), and PEDOT-Al 0.75 mC 200 °C (16–18 nm). These results are in line with the thickness of the electrodeposited polythiophene-based MTHs [40]. In addition, it was observed that the annealing temperature of 240 °C does not affect the thickness of PEDOT-Al 0.50 mC, thus polymer shrinkage due to thermal degradation was discarded.

The morphology of electrodeposited bilayers was compared with that of bilayers obtained by physical vapor deposition (PVD). For this purpose, a ~2 nm Al layer was thermally evaporated on PEDOT:PSS-Cl and annealed at 200° C. The thickness (16–18 nm) and homogeneity of the resulting PVD bilayer suggest that the electrochemical process offers similar quality and film thickness control as PVD. Regarding the perovskite layer, cross-sectional SEM images show similar monolithic vertical grains for all variations (Figure S3), which is beneficial for cell performance and stability [41].

By AFM imaging, it was observed that the ITO substrate has a root mean square (RMS) roughness of 1.7 nm (Figure S5). After electrodepositing the HTMs, the RMS roughness increased by a similar value for all variations (around 3 nm), even for the inorganic layer by PVD. This indicates that the homogeneity of the electrodeposited Al_2_O_3_ is comparable to that obtained by PVD. An important advantage of the electrodeposition of polymers and oxides is the low film thickness and roughness that can be obtained [22,40,42]. As for hydrophilicity, contact angle measurements showed that Al_2_O_3_ has a hydrophobic nature (Figure S6a–f). Hence, when used to modify PEDOT:PSS-Cl films, it causes the polymer to become more hydrophobic. This could explain the slight improvement in the shelf stability of the unencapsulated PEDOT-Al devices compared to PEDOT:PSS-Cl.

### 3.2. Spectroscopic and Electrochemical Characterization

Additional spectroscopic characterizations were performed to identify the reasons why PEDOT-Al is a better HTM than PEDOT:PSS-Cl.

**Figure 4:**
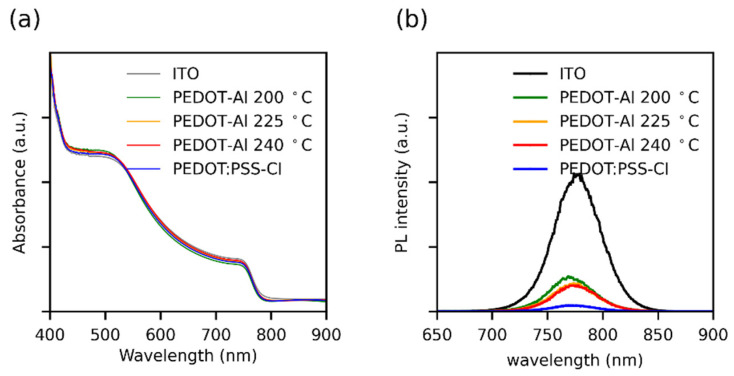
(**a**) UV-Vis absorption spectra and (**b**) steady-state photoluminescence spectra of perovskite films deposited over different HTMs. The UV-Vis spectra were taken using an ITO substrate as a reference.

Considering that the perovskite layer had the same thickness in all devices, it was confirmed by UV-visible absorption spectroscopy that perovskite films deposited on the different substrates show no differences in light absorption (Figure 4a). Stationary photoluminescence (PL) showed that all HTMs induce strong PL quenching compared to perovskite deposited on ITO (Figure 4b), and PEDOT:PSS-Cl showed the highest PL quenching. Although this seems to go against the higher J_SC_ found for PEDOT-Al bilayers, it has been demonstrated that, when the most intense PL corresponds to the most efficient device, it is considered indicative that the HTM modification induces changes in recombination phenomena from a non-radiative to a radiative process [43,44]. In the PEDOT-Al bilayers, Al_2_O_3_ could produce this effect by passivating the non-coordinated Pb^2+^ ions on the perovskite surface, thus reducing the trap states responsible for non-radiative recombination, which correlates with the V_oc_ enhancement [18,43,44,45].

As previously mentioned, one of the reasons for the improved performance of PEDOT-Al devices could be the presence of an energy barrier that increases charge selectivity. In order to test this, an estimation of the HOMO level was performed by cyclic voltammetry. However, it is clarified that these values are used for comparative purposes between variations, since this characterization is carried out in liquid media, which is drastically different from the operating conditions of solar cells. Even so, this technique is widely used, as it correctly reproduces the trends of other accurate, but more expensive, photoemission-based techniques [46,47,48,49,50]. Ferrocene was used as a reference for the vacuum level, and the HOMO level was calculated using the onsets of the oxidation potentials (Figure S7 and Table S2). The HOMO level values obtained were 5.06 eV for PEDOT:PSS-Cl and 5.10 eV for all PEDOT-Al bilayers, demonstrating the additional energy barrier imposed by the inorganic layer. These results concur with previous works on ALD-deposited Al_2_O_3_. For example, it was reported that the Al_2_O_3_ layer on PEDOT-Al has a passivating effect and imposes an energy barrier that increases charge selectivity, which results in increased PCE, V_oc_, and J_sc_ [18,51]. Similarly, when ALD-Al_2_O_3_ is used as an intermediate layer between perovskite and Spiro-OMeTAD HTM, there is an increase in PCE from 15.1% to 18.0%. The ability to use ALD on any surface is a remarkable advantage, as it allows Al_2_O_3_ to be deposited in direct structure devices in which the hydrophobic nature of this layer is an effective barrier between the ambient humidity and the perovskite layer. As a result, the device stability is notoriously increased, whereby solar cells have been reported to maintain 60–70% of their original PCE even after 70 days of storage [17,52].

e-Beam processed interlayer materials between the HTM and the perovskite have also showed similar improvements to those reported here. On MAPbBr_3_ perovskite devices, the HOMO level of NiOx HTM have been improved by using an overlayer of e-Beam MoOx. The efficiency goes from 2.79% for NiOx to 5.2% for the NiOx/e-Beam MoOx solar cells. The MAPbBr_3_ perovskite shows relatively low PCE nonetheless it shows promising high V_oc_ values, which can be useful for applications such as solar driven water electrolysis, photocatalysis, and multijunction solar cells. A remarkable 1.653 V V_oc_ (10.08% PCE) has been achieved by using interlayers at both sides of the perovskite, e-Beam MoOx and ALD ZrO_2_ for the HTM and ETM interfacial modification [53].

**Figure 5:**
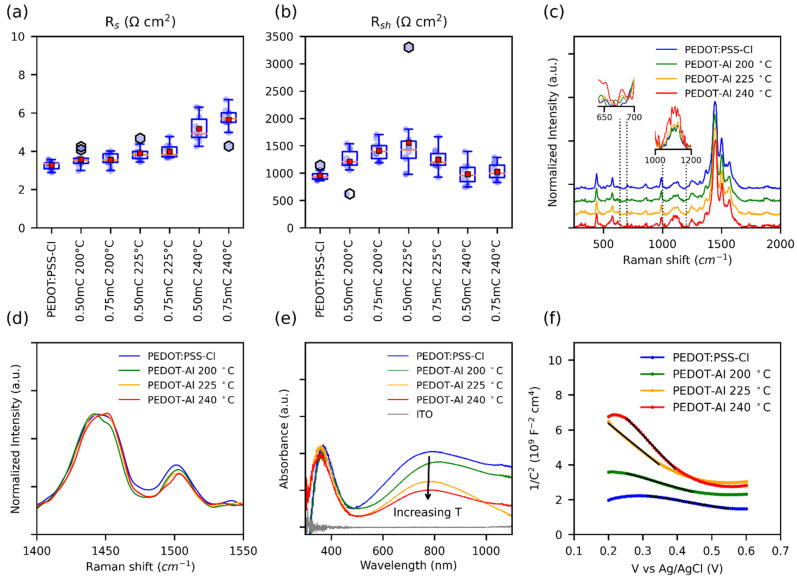
(**a**) Box plot of R_s_ and (**b**) R_sh_ of perovskite solar cells with different HTMs, values were estimated from the slopes of the JV curves. Note: PEDOT-Al was removed from the labels. (**c**) Normalized Raman spectra, insets show the Raman intensity comparison of the normalized data. (**d**) Intensities in the range of the main peak. (**e**) UV-Vis spectra. (**f**) Mott–Schottky curves taken in 0.1 M Na_2_SO_4_ with an applied DC voltage from 0.2 V to 0.6 V; the solid black lines represent the fitted data used to extract the slopes.

To understand the noticeable decrease in FF with the annealing temperature of 240 °C (Figure 1d), the series (R_s_) and shunt resistances (R_sh_) of the solar cells were plotted (Figure 5a,b). For optimum FF, the R_s_ should be as low as possible and R_sh_ should be high. Nevertheless, it has been observed that, in perovskite solar cells, the FF and therefore the PCE are particularly affected by R_s_ [54], and the influence of shunt resistance (R_sh_) is only noticeable below 1000 Ω cm^2^. Ahmed et al. found similar results, where a R_s_ as low as 6 Ω cm^2^ can result in a poor FF (67%) [55]. Therefore, the decrease in FF for the annealing temperature of 240 °C can be attributed to the increase in R_s_. This resistance can be caused by ohmic elements, such as metallic contact, ETM, HTM, or conductive substrate [54]. Since the HTM is the layer that is varying, the observed changes in R_s_ were assigned to the R_s_ of this material. Moreover, the annealing process could induce thermal degradation of PEDOT, which decreases its conductivity [56].

Raman spectroscopy characterization of PEDOT:PSS-Cl and PEDOT-Al 0.50 mC was performed in search of thermal degradation signals that would explain the increase in R_s_ (Figure 5c). All the samples analyzed showed the characteristic PEDOT:PSS signals at 440 cm^−1^, 575 cm^−1^, 990 cm^−1^, 1252 cm^−1^, 1366 cm^−1^, 1445 cm^−1^, 1500 cm^−1^, and 1570 cm^−1^ [57,58]. Thus, no apparent degradation was found even at the annealing temperature of 240 °C. Nonetheless, the relative Raman intensities were compared and it was noticed that the signals around 650 cm^−1^, 680 cm^−1^, and between 1000 cm^−1^ and 1200 cm^−1^ increased with annealing temperature (insets Figure 5c). The increase in these signals has been related to low-chain planarity, and structural defects in the polymeric chains due to the formation of side groups during overoxidation [59,60]. The degradation of PEDOT by overoxidation has been linked to photo-oxidation, electrochemical overoxidation, and thermal degradation. These processes point to a degradation mechanism involving the oxidation of the sulfur atom in the thiophene ring creating a sulfoxide side group (R-SO-R), which is then oxidized to sulfone (R-SO_2_-R) [56,61]. Signals from these groups are expected in the range of 1000–12,000 cm^−1^ [62,63,64]. In the case of severe overoxidation, oxidative elimination of SO_2_ opens the thiophene ring leading to the formation of carbonyl groups, and even polymer chain breakage may occur with the appearance of carboxyl groups [56,61]. Since for the annealing temperature of 240 °C Raman spectra showed no evidence of carbonyl or carboxyl formation, the observed thermal degradation could be an initial stage of overoxidation, originating sulfoxide and sulfone side groups [61].

In general, the conductivity in conjugated polymers, including PEDOT, is limited by interchain charge transport, since intrachain transport along the polymer backbone is much faster. A low π–π stacking distance is required to increase interchain transport and overcome charge localization within the polymer chain [65]. The molecular π–π stacking distance depends on chain planarity and interchain packing. This is notably affected by the thermal degradation that generates side groups in the polymer backbone, and thus impairs the conductivity. In addition, side groups also reduce the chain conjugation length and increase charge localization, and, as a result, charge transport is restricted [65,66,67]. This should especially be the case for PEDOT-Al deposits annealed at 240 °C, as Raman spectra suggest more side groups at this temperature. The shift of the main peak (from 1445 cm^−1^ to 1451 cm^−1^) at the annealing temperature of 240 °C suggests a structural transformation of the PEDOT chains from quinoid to benzoid (Figure 5d) [68]. The quinoid conformation with its flat and straight structure favors π–π interactions between chains, whereas the benzoid structure prefers a coiled arrangement of the PEDOT chains. Therefore, at 240 °C, the π–π staking distance should increase, and the coiling of the chains would affect intrachain mobility and polymer packing. All of the above leads to limited intrachain and interchain transport, which contributes to the pronounced increase in polymer resistance [68,69].

Polymer degradation was also evidenced from the UV-Vis spectra of PEDOT:PSS-Cl and PEDOT-Al 0.50 mC. In this paper, a noticeable decrease in absorption was observed around 800 nm with increasing annealing temperature (Figure 5e). This signal has been related to electronic transitions due to the presence of polarons in the polymer. Therefore, in line with the increase in R_s_, the annealing temperature affects the charge carriers in the polymer [58]. Similar Raman and UV-Vis results were found for PEDOT-Al 0.750 mC (Figure S8).

To confirm the decrease in polaronic states, a Mott–Schottky analysis was performed to determine the charge carrier density in HTMs. In conducting polymers, this is challenging as the doping level may change due to the potentials applied during the measurements. To extract information that resembles the original state of the HTMs, a low polarization electrochemical window was chosen. Figure 5f shows the results: in this plot, the slope of the linear section is inversely proportional to the charge carrier density (Table S3) [70,71]. As expected, the PEDOT:PSS-Cl and PEDOT-Al 0.50 mC 200 °C films have the highest charge carrier density. Moreover, it can be observed that the carrier density decreases as the annealing temperature increases. This reduction of charge carriers (polarons) in this polymer has been related to the formation of bonds between the chlorides (Cl^−^) and positive charges on the polymer chains, and to the recombination with radicals generated during degradation [56,59,72]. Since reverse and forward sweeps showed similar results, it follows that the effect of doping was significantly reduced under these experimental conditions. Overall, Raman, UV-Vis, and Mott–Schottky characterizations reveal that the mechanism affecting the conductivity of PEDOT is accentuated at 240 °C and contributes to the noticeable increase in R_s_, resulting in low FF and limiting the PCE of the PEDOT-Al devices.

**Figure 6:**
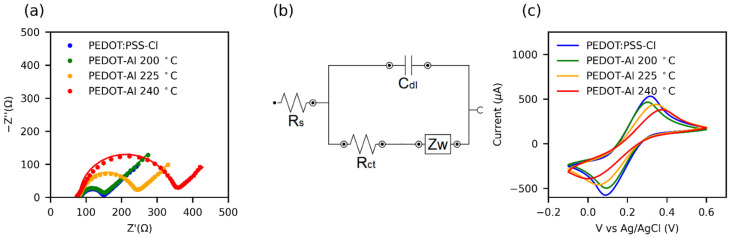
(**a**) Nyquist plots from electrochemical impedance spectroscopy (EIS) of HTMs in 0.1 M KCl + 5 mM Fe(CN)_6_^3−/4−^ aqueous solution. The solid lines represent the fit of the EIS data using the equivalent circuit shown in (**b**). (**c**) Cyclic voltammograms at 100 mV s^−1^ in the same solution.

The effect of thermal degradation on the HTMs was also investigated through electrochemical impedance (EIS) and cyclic voltammetry (Figure 6a–c). Table S4 presents the charge transfer resistance (R_ct_) values obtained by fitting the results to the equivalent circuit in Figure 6b. It was found that, for the annealing temperature of 200 °C, PEDOT-Al had almost the same R_ct_ as pristine PEDOT:PSS-Cl, and increasing the annealing temperature also increased the R_ct_. In conducting polymers, the R_ct_ is closely related to the microstructure. Thus, the symmetry and reversibility of the redox reaction depend on the overlap of the density of states (DOS) of the polymer and the redox probe, whereby a highly organized microstructure results in a DOS of the polymer that facilitates reversible charge transfer [73].

Cyclic voltammetry results for the ferricyanide redox reaction (Figure 6c) show that the peak separation (ΔEp) increases with the increase in the annealing temperature of HTMs. In turn, the redox currents decrease and the peaks are less defined. This may be attributed to a structural disorder caused by the thermal degradation of the polymer [74]. Moreover, some reports relate the increased R_ct_ of PEDOT to its overoxidation [75] and the presence of side groups in the polymer chain [67]. Consequently, for annealing processes, a low R_ct_ value can be taken as indicative of a high-quality conductive polymer for solar cell applications.

### 3.3. Impedance Spectroscopy Analysis

**Figure 7:**
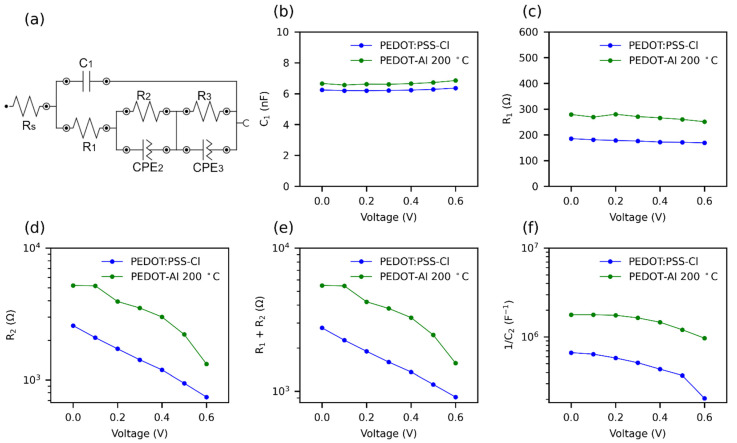
Effect of PEDOT-Al 0.50 mC 200 °C on the parameters of impedance spectra at various applied voltages, under 1 sun illumination. (**a**) Equivalent circuit for fitting impedance spectra. R: resistance; C: capacitance; and CPE: constant phase elements. (**b**) C_1_, (**c**) R_1_, (**d**) R_2_, (**e**) R_1_ + R_2_, and (**f**) 1/C_2_; C_2_ was extracted from CPE_2_.

Finally, impedance spectroscopy (IS) was performed to compare the solar cells based on PEDOT:PSS-Cl and PEDOT-Al 0.50 mC 200 °C. In both cases, the Nyquist plots show two well-defined semicircles followed by considerable noise, which takes the form of a third semicircle as the applied potential increases (Figure S9). Some investigations have linked the third semicircle to ionic motion in perovskite [76,77], and noise to ionic motion and reactivity of metal contacts [78]. Although the interpretation of the impedance spectra is still a matter of debate, since there is no general model for perovskite solar cells, the equivalent circuit in Figure 7a was used to fit the data [78,79].

There is some consensus that the R_s_ corresponds to losses due to ohmic resistance in the cell and wiring [79]. Furthermore, in the impedance spectrum (IS), the first arc at high frequencies is attributed to the charge transfer resistance and recombination resistance (R_1_) [39]. The capacitance of this arc is of the geometrical type and is due to the perovskite material (C_1_) [80]. The second arc at intermediate frequencies is due to the recombination resistance R_2_, although it is usual to add R_1_ and R_2_ to estimate this resistance [39,79]. The capacitance of the second arc C_2_ represents the charge accumulation at the cell interfaces, which causes defects in the perovskite leading to recombination [44,80]. This capacitance increases rapidly near the maximum power point (MPP) of the solar cell. It is common to plot 1/C_2_ versus applied potential, as the trend resembles JV curves [44]. Due to noise, the parameters of the third arc were not taken into account. The obtained IS parameters are shown in Figure 7b–f. The Rs (9 Ω) and C1 presented similar value with both HTMs (PEDOT:PSS-Cl, PEDOT:Al). The incorporation of Al_2_O_3_, which is a dielectric material, justifies the slight increase in capacitance (from 6.2 to 6.7 nF). It is also observed that R_1_+ R_2_ was higher for PEDOT-Al, showing that the electrochemical incorporation of the Al_2_O_3_ layer markedly increases the recombination resistance (Figure 7e).

A solar cell with a high recombination resistance will present a higher current density at an applied potential, leading to a higher V_oc_ [31]. The recombination resistance of PEDOT-Al decreased faster initially, but maintained a higher value compared to PEDOT:PSS-Cl, hence the increased current density in the 0 to 0.6 V range and the higher V_oc_ (Figure 2a). Therefore, this suggests that the use of PEDOT-Al helps to suppress charge recombination. The increase in recombination resistance is also confirmed by the C_2_ capacitance values extracted from CPE_2_. Figure 7f shows the variation of 1/C_2_ with voltage for the two HTMs. A slight decrease is observed for PEDOT-Al, while for PEDOT:PSS-Cl the decrease is larger and drops abruptly at 0.5 V. A high and nearly constant 1/C_2_ value represents better current density and steady-state PCE for PEDOT-Al. In contrast, PEDOT:PSS-Cl has a lower 1/C_2_ value leading to a lower current density JV curve in the 0 to 0.6 V range.

Impedance spectroscopy generates results more similar to steady-state results than JV curves. This is because a constant potential is applied for several minutes, whereas the JV uses a fast potential sweep. Therefore, the decrease of 1/C_2_ at 0.5 V for PEDOT:PSS-Cl can be attributed to the inability of this device to maintain the current density at a constant potential above 0.5 V, thus, the lower steady-state efficiency compared to the JV PCE, since the steady-state measurements were conducted at 0.75 V [44]. It is suggested that ionic charge accumulation at the PEDOT:PSS-Cl/perovskite interface leads to a high defect density in the perovskite, whereas PEDOT-Al decreases the presence of these defects (lower C_2_ values).

This is supported by the Mott–Schottky analysis (Figure S2), which showed that ions can easily penetrate the PEDOT:PSS-Cl layer causing doping/dedoping of the polymer [37]. In contrast, in PEDOT-Al, the insertion of halide ions is restricted [81], so fewer of these ions can migrate and leave the perovskite.

## 4. Conclusions

This study presented the fabrication and evaluation of inverted perovskite solar cells, using as HTM a PEDOT-Al_2_O_3_ bilayer obtained by electrochemical methods. It was shown that the incorporation of electrochemical Al_2_O_3_ improves the charge selectivity, passivates the perovskite, and prevents doping of the polymer. As a result, the efficiency of the solar cell increased from 12.1% to 14.3%. Notably, it was observed that the steady-state efficiency of the PEDOT- Al_2_O_3_ cell is higher than the efficiency determined from JV curves, whereas without Al_2_O_3_ it produces 92% of its JV efficiency in steady-state tests. The addition of electrochemical Al_2_O_3_ also increased the cell stability by 2% during a 45 min continuous operation test.

The electrochemical Al_2_O_3_ deposition methodology used in this work is much simpler and more affordable than the atomic layer deposition method. Nevertheless, special attention must be given to the annealing step as it was found that a high temperature treatment degrades the PEDOT (sulfur oxidation, change from quinoid to benzoid, and decrease in polarons), reducing its conductivity and cell FF. However, this effect was minimized by using an annealing temperature of 200 °C.

The results presented in this work aim to contribute to the development of advanced HTMs in perovskite solar cells based on electrochemical techniques.

## Data Availability

The data presented in this study are available on request from the corresponding author.

## Supplementary Materials

The following are available online at https://www.mdpi.com/article/10.3390/polym13234162/s1: Table S1: Mean and standard deviation (SD) of the photovoltaic parameters of solar cells containing different HTMs; Figure S1: Evolution of (a) PCE, (b) Jsc, (c) Voc, and (d) FF of the devices. The dots represent the mean value, and the bars show the standard deviation, 4 devices for each variation. The devices were stored under a N_2_ atmosphere and exposed to 70% RH during the measurements; Figure S2: Mott–Schottky plots of the ITO/HTMs electrodes in 0.1 M NaCl. (a) and (b) are replicate of PEDOT:PSS-Cl films exposed to consecutive Mott–Schottky measurements, and numbers represent scan order. (c) reverse and (d) forward plots of PEDOT-Al 0.50 mC, and (e) reverse and (f) forward plots of PEDOT-Al 0.75 mC. The reverse scan for PEDOT:PSS-Cl is plotted for comparison; Figure S3: Cross-section SEM images of perovskite solar cells fabricated with (a) PEDOT:PSS-Cl, (b) PEDOT-Al 0.5 mC 200 °C, and (c) PEDOT:PSS-Cl with 2 nm Al PVD. The evaporated Al layer was submerged in water for 7 h and annealed for 1 h at 200 °C. Left, secondary electrons (SE), and right, backscattered electrons (BSE); Figure S4: Cross-section SEM images of perovskite films deposited on various PEDOT-Al substrates. Backscattered electrons (BSE); Figure S5: (a–f) AFM topographic images and RMS roughness of ITO, PEDOT:PSS-Cl, and PEDOT-Al substrates; Figure S6: (a–f) Static contact angle of water over different materials deposited over ITO; Figure S7: (a) Cyclic voltammetry of ferrocene reference. (b–d) Cyclic voltammetry of PEDOT-Al 0.50 mC HTMs. In (b), the PEDOT:PSS-Cl and PEDOT-Al 0.5 mC 200 °C films are compared, and the Vox onset was 0.68 V and 0.72 V, respectively; Table S2: HOMO levels estimated by cyclic voltammetry; Figure S8: (a) normalized Raman spectra of reference PEDOT:PSS-Cl and PEDOT-Al 0.75 mC bilayers. The insets show the Raman intensity comparison of the normalized data, in (b) the intensities in the range of the main peak are displayed, and (c) shows the corresponding UV-Vis spectra; Table S3: ND Charge carrier density from Mott–Schottky analysis with forward and reverse scans on each film; Table S4: Charge transfer resistance extracted from the EIS fitting; Figure S9: Nyquist plots for the solar cells at different applied DC voltage (a–g). The continuous lines represent the fitting of the IS data. The insets show a magnification of the data.
