# Supplementary material for: Electrodeposited PEDOT:PSS-Al2O3 Improves the Steady-State Efficiency of Inverted Perovskite Solar Cells
Source: Polymers (Basel). 2021 Nov 28;13(23):4162. doi: 10.3390/polym13234162 (PMC8659661; doi:10.3390/polym13234162)
Supplement: Supplementary file 1 [file polymers-13-04162-s001.zip › polymers-1444128-supplementary.pdf]

Electrodeposited PEDOT:PSS-Al_2O_3 Improves the Steady-State Efficiency of Inverted Perovskite Solar Cells

Eider A. Erazo[1], Martín Gómez[2], Leonardo Rios[3], Edgar J. Patiño[3], María T. Cortés[1,*], Pablo Ortiz[2]

[1] Departamento de Química, Universidad de los Andes, Bogotá D.C. 111711, Colombia; ea.erazo@uniandes.edu.co (E.A.E.); marcorte@uniandes.edu.co (M.T.C.)
[2] Departamento de Ingeniería Química, Universidad de los Andes, Bogotá D.C. 111711, Colombia; m.gomezd@uniandes.edu.co (M.G.); portiz@uniandes.edu.co (P.O.)
[3] Superconductivity and Nanodevices Laboratory, Departamento de Física, Universidad de los Andes, Bogotá D.C. 111711, Colombia; l.rios954@uniandes.edu.co (L.R.); epatino@uniandes.edu.co (E.J.P.)
* Correspondence: marcorte@uniandes.edu.co (M.T.C.)

Supplementary material

Table S1. Mean and standard deviation (SD) of the photovoltaic parameters of solar cells containing different HTMs.

HTM	PCE (%) (Mean ± SD)	Jsc (mA cm^{-2}) (Mean ± SD)	Voc (V) (Mean ± SD)	FF (%) (Mean ± SD)
PEDOT:PSS-Cl	11.5 ± 0.6	16.3 ± 0.7	0.918 ± 0.009	77 ± 1
PEDOT-Al 0.50mC 200°C	13.6 ± 0.5	19.6 ± 0.3	0.951 ± 0.016	73 ± 2
PEDOT-Al 0.75mC 200°C	13.9 ± 0.5	19.8 ± 0.5	0.957 ± 0.015	73 ± 1
PEDOT-Al 0.50mC 225°C	13.2 ± 0.7	18.8 ± 1.3	0.948 ± 0.013	74 ± 2
PEDOT-Al 0.75mC 225°C	13.1 ± 0.5	19.1 ± 0.4	0.950 ± 0.014	73 ± 2
PEDOT-Al 0.50mC 240°C	12.1 ± 0.6	19.2 ± 0.4	0.936 ± 0.018	67 ± 2
PEDOT-Al 0.75mC 240°C	12.3 ± 0.5	19.7 ± 0.4	0.955 ± 0.007	65 ± 2

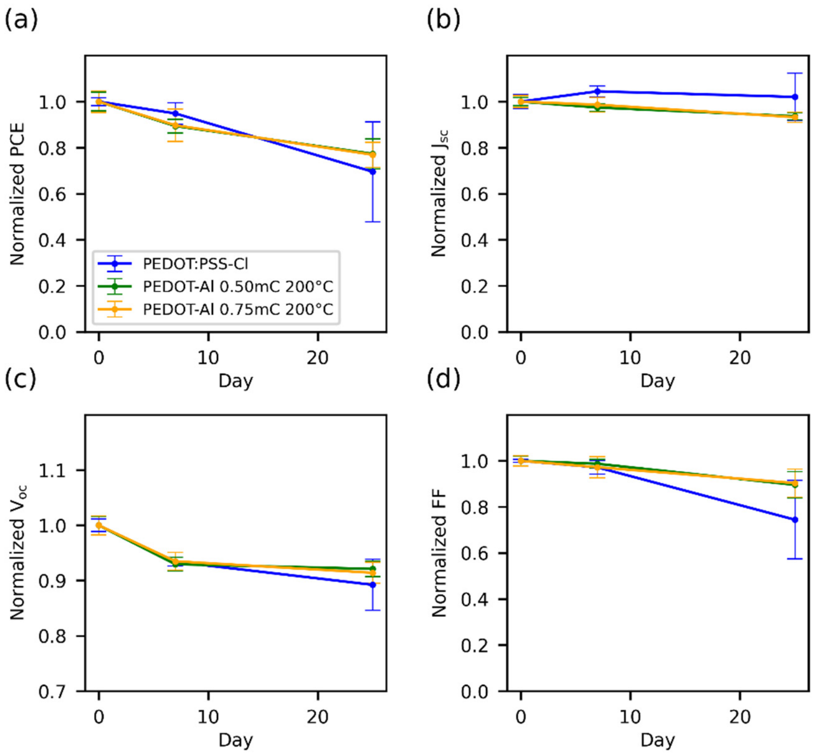

Figure S1. Evolution of (a) PCE, (b) Jsc, (c) Voc, and (d) FF of the devices. The dots represent the mean value, and the bars show the standard deviation, 4 devices for each variation. The devices were stored under N_2 atmosphere and exposed to 70% RH during the measurements.

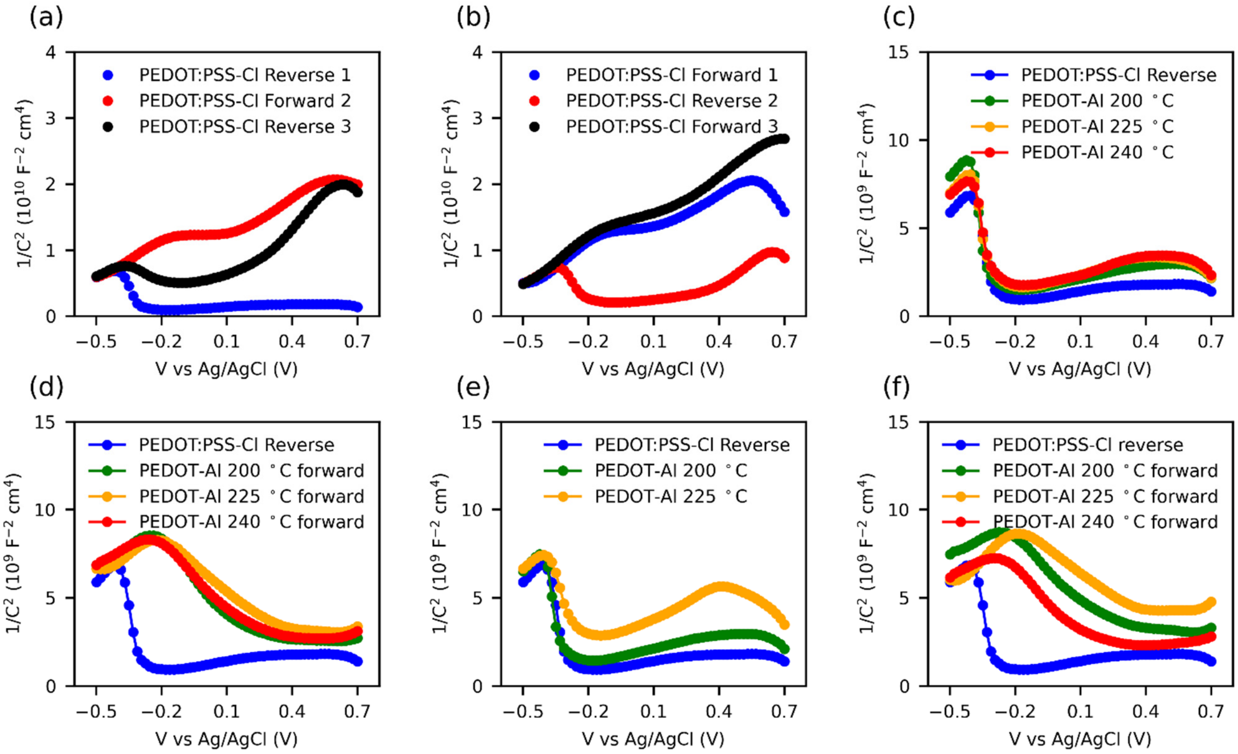

Figure S2. Mott-Schottky plots of the ITO/HTMs electrodes in 0.1 M NaCl. (a) and (b) are replicate of PEDOT:PSS-Cl films exposed to consecutive Mott-Schottky measurements, numbers represent scan order. (c) reverse and (d) forward plots of PEDOT-Al 0.50 mC (e) reverse and (f) forward plots of PEDOT-Al 0.75 mC, the reverse scan for PEDOT:PSS-Cl is plotted for comparison.

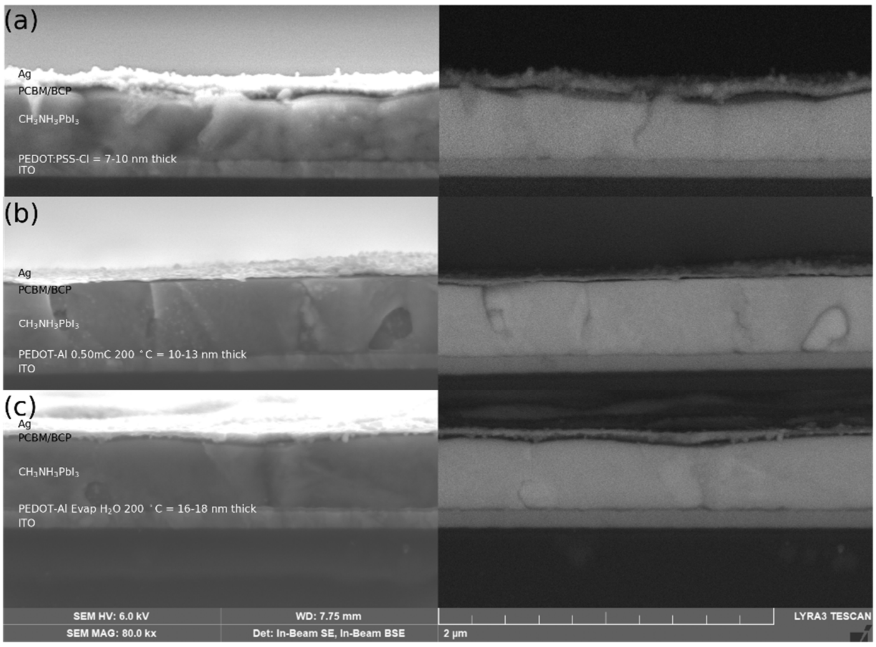

Figure S3. Cross-section SEM images of perovskite solar cells fabricated with (a) PEDOT:PSS-Cl, (b) PEDOT-Al 0.5 mC 200 °C, and (c) PEDOT:PSS-Cl with 2 nm Al PVD. The evaporated Al layer was submerged in water for 7 h and annealed for 1 h at 200 °C. Left secondary electrons (SE), right backscattered electrons (BSE)

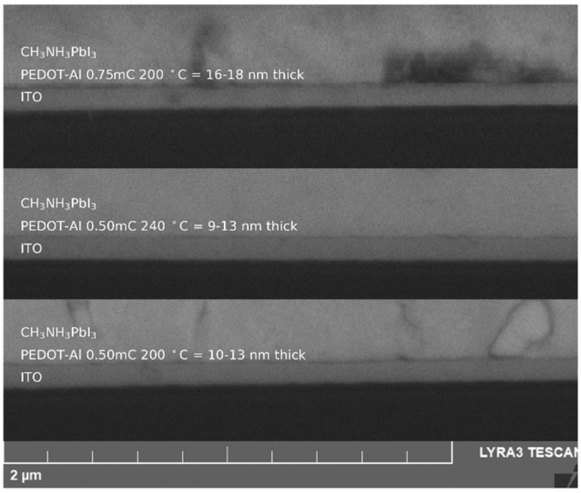

Figure S4. Cross-section SEM images of perovskite films deposited on various PEDOT-Al substrates. Backscattered electrons (BSE).

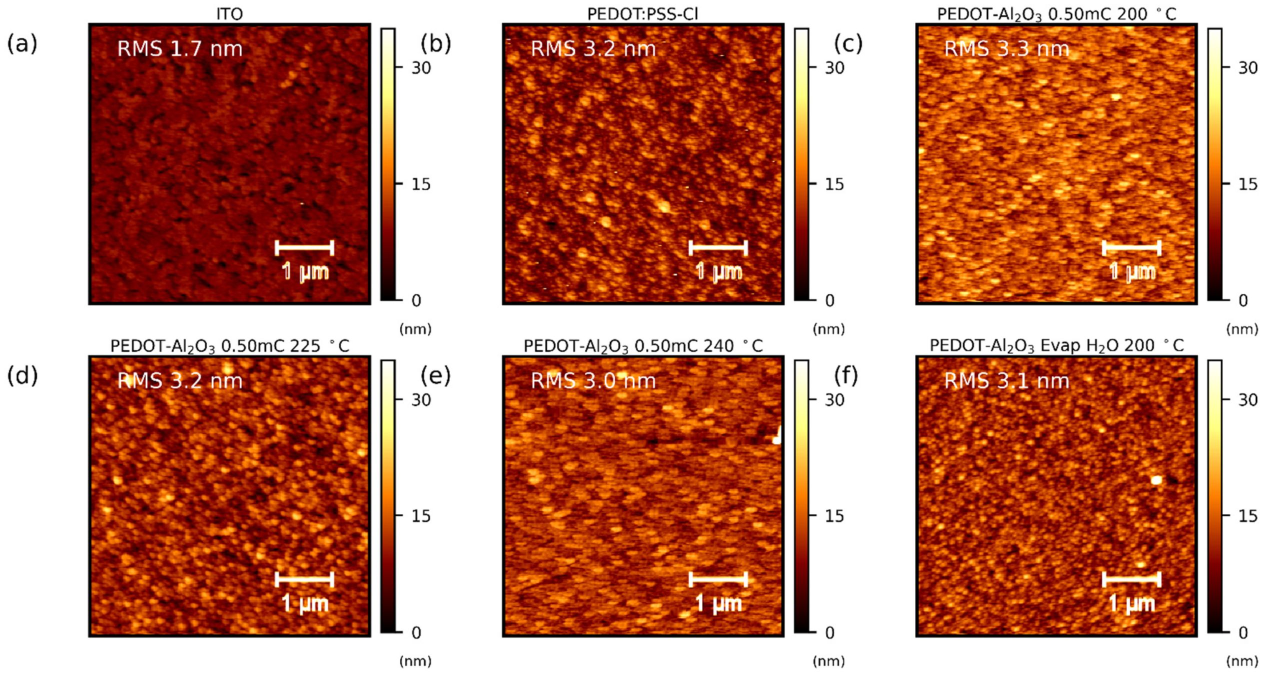

Figure S5. (a-f) AFM topographic images and RMS roughness of ITO, PEDOT:PSS-Cl, and PEDOT-Al substrates.

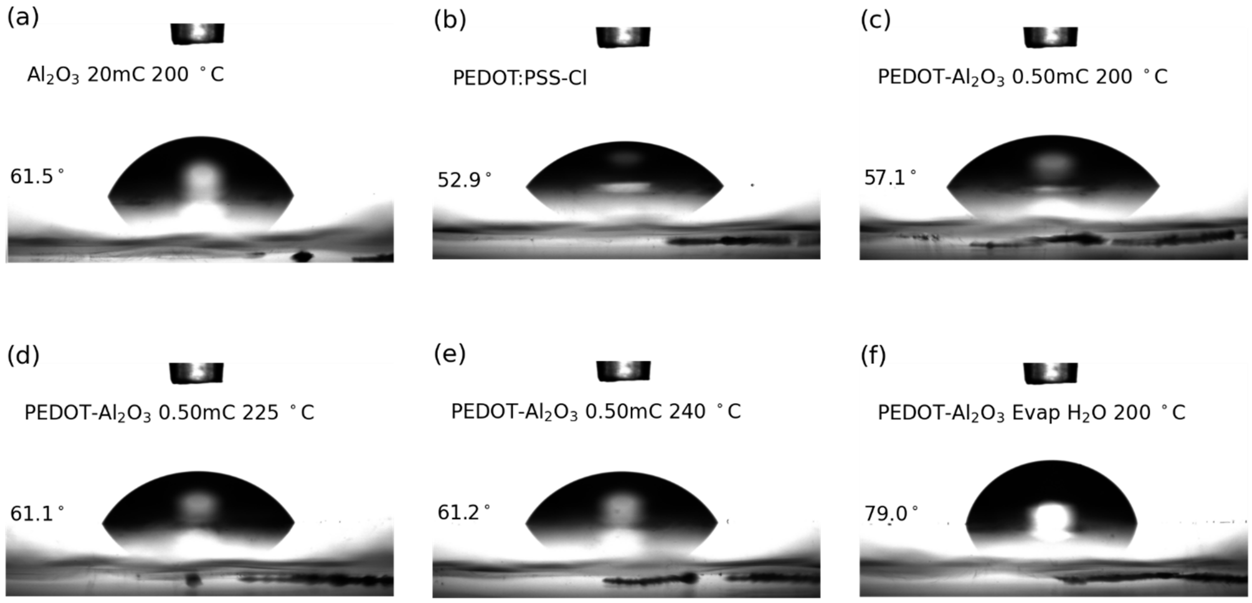

Figure S6. (a-f) Static contact angle of water over different materials deposited over ITO.

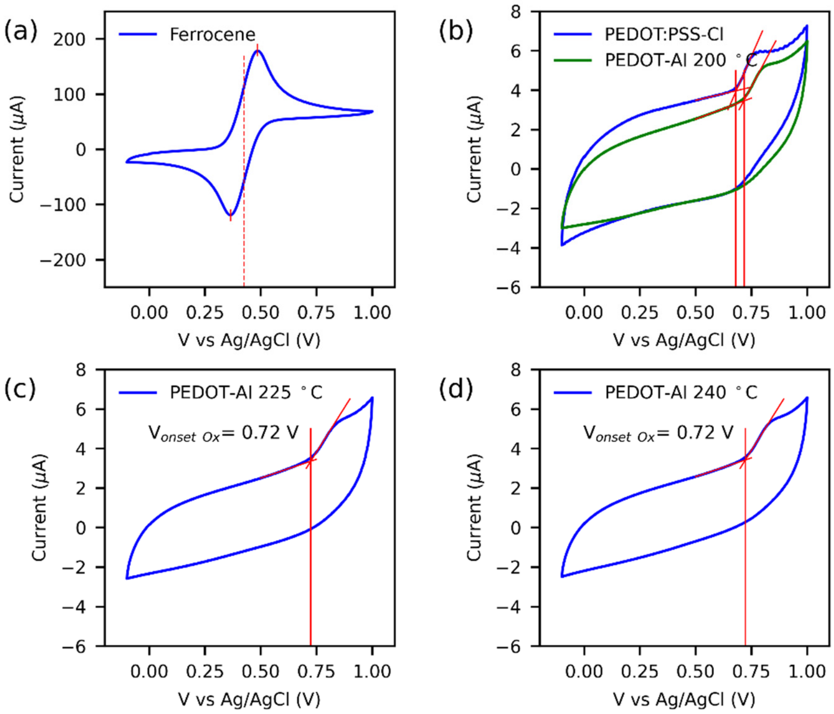

Figure S7. (a) Cyclic voltammetry of ferrocene reference. (b-d) Cyclic voltammetry of PEDOT-Al 0.50 mC HTMs. In (b) PEDOT:PSS-Cl and PEDOT-Al 0.5mC 200 °C films are compared, the Vox onset was 0.68 and 0.72 V respectively.

$$HOMO = -(V_{ox} + 4.80 - V_{ox,ferrocene})eV \qquad (1)$$

Table S2. HOMO levels estimated by cyclic voltammetry.

Variation	Vox(V)	HOMO (eV)
PEDOT:PSS-Cl	0.68	-5.06
PEDOT-Al 0.50mC 200 °C	0.72	-5.10
PEDOT-Al 0.50mC 225 °C	0.72	-5.10
PEDOT-Al 0.50mC 240 °C	0.72	-5.10
Ferrocene	0.42	

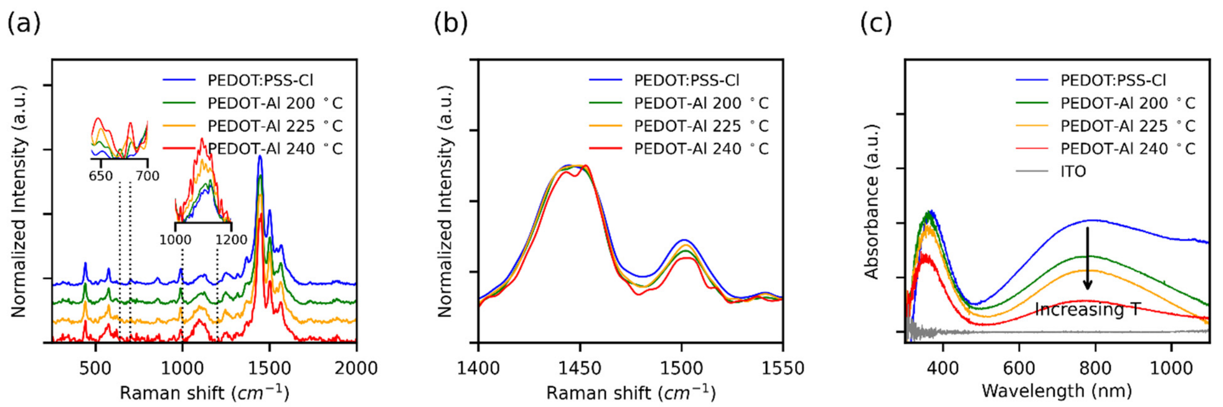

Figure S8 . (a) normalized Raman spectra of reference PEDOT:PSS-Cl and PEDOT-Al 0.75 mC bilayers, the insets show the Raman intensity comparison of the normalized data, in (b) the intensities in the range of the main peak are displayed, and (c) shows the corresponding UV-Vis spectra.

In the Mott-Schottky analysis, a plot of C^{-2} versus V yields a straight line whose slope allows calculation of the charge carrier density N_D.

$$N_D = \frac{2}{e\varepsilon_r\varepsilon_0 Slope} \quad (2)$$

Where e is de electron charge, ε_r is the dielectric constant and ε_0 is the vacuum permittivity, the value of ε_r value for PEDOT:PSS-Cl was chosen as ~2.5,[1]. The immersed area of the electrodes was 0.52 cm^{-2}, e = 1.602×10-19 C and ε_0 is 8.8541×10-12Fm^{-1}.[2]

Table S3. ND Charge carrier density from Mott-Schottky analysis whit forward and reverse scans on each film.

Variation	Forward Slope (10^9 F^{-2} cm^4V^{-1})	Forward ND (cm^{-3})	Reverse Slope (10^9 F^{-2} cm^4V^{-1})	Reverse ND (cm^{-3})
PEDOT:PSS-Cl	3.26	$1.7x10^{17}$	7.15	$7.9x10^{16}$
PEDOT-Al 0.50mC 200°C	5.65	$1.0x10^{17}$	8.14	$6.9x10^{16}$
PEDOT-Al 0.50mC 200°C	16.05	$3.5x10^{16}$	17.68	$3.2x10^{16}$
PEDOT-Al 0.50mC 225°C	20.63	$2.7x10^{16}$	24.47	$2.3x10^{16}$

Table S4. Charge transfer resistance extracted from the EIS fitting.

Variation	PEDOT:PSS-Cl	PEDOT-Al 200 °C	PEDOT- Al 225 °C	PEDOT- Al 240 °C
Rct (Ω)	56.12	57.04	148.67	257.22

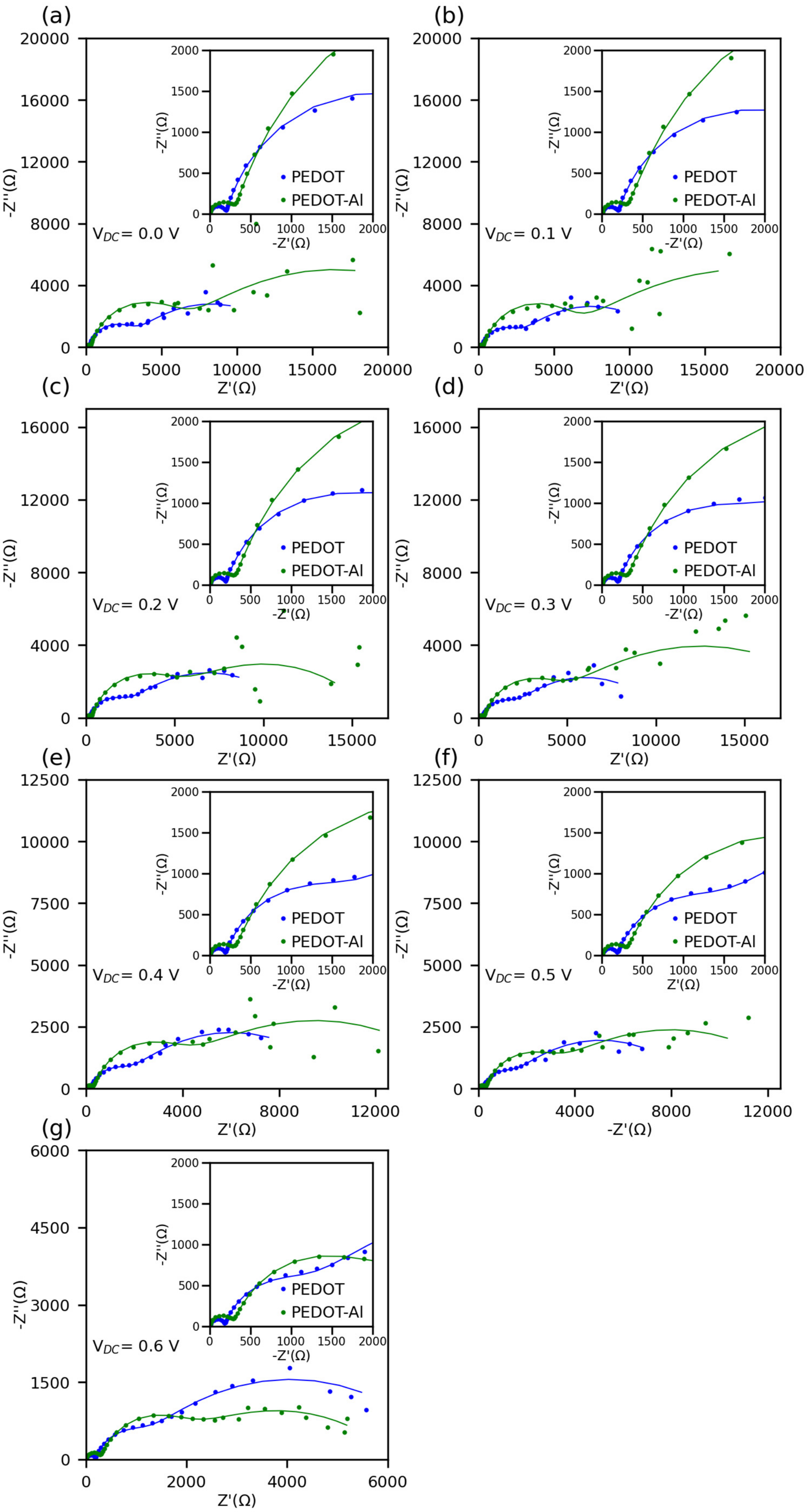

Figure S9. Nyquist plots for the solar cells at different applied DC voltage (a-g). The continuous lines represent the fitting of the IS data. The insets show a magnification of the data.

References

1. Muckley, E.S.; Jacobs, C.B.; Vidal, K.; Mahalik, J.P.; Kumar, R.; Sumpter, B.G.; Ivanov, I.N. New Insights on Electro-Optical Response of Poly(3,4-ethylenedioxythiophene):Poly(styrenesulfonate) Film to Humidity. *ACS Appl. Mater. Interfaces* **2017**, *9*, 15880-15886, doi:10.1021/acsami.7b03128.
2. Sahoo, P.P.; Zoellner, B.; Maggard, P.A. Optical, electronic, and photoelectrochemical properties of the p-type Cu3-xVO4 semiconductor. *J. Mater. Chem. A* **2015**, *3*, 4501-4509, doi:10.1039/c4ta04876h.
